# Multidimensional factors shaping older persons’ resilience to floods in Madura Island

**DOI:** 10.4102/jamba.v17i1.1755

**Published:** 2025-06-17

**Authors:** Hijrah Saputra, Prasetyo W. Iswara, Nik Norliati Fitri Md Nor, Fadly Usman

**Affiliations:** 1 Department of Disaster Management, Postgraduate School, Universitas Airlangga, Surabaya, Indonesia; 2 Department of Business, Faculty of Vocational Studies, Universitas Airlangga, Surabaya, Indonesia; 3 Department of Geography, School of Distance Education, Universiti Sains Malaysia, Penang, Malaysia; 4 Department of Urban and Regional Planning, Faculty of Engineering, Brawijaya University, Malang, Indonesia

**Keywords:** disaster risk reduction, flooding, Madura Island, multidimensional analysis, older persons’, vulnerability

## Abstract

This study examines the multidimensional factors that influence the resilience of older persons in responding to and recovering from flood hazard in the Madura Island, Indonesia. Using Spearman correlation analysis and Principal Component Analysis (PCA), the study evaluates key demographic, health, risk perception, institutional and attitudinal characteristics that shape disaster resilience. A structured survey was conducted with 399 older persons across four districts: Bangkalan, Sampang, Pamekasan and Sumenep. The findings indicate that age, health status, prior flood experience, institutional support and disaster preparedness play a significant role in shaping resilience. Critical indicators include gender, the use of mobility aids, need for activity assistance, access to emergency services, physical limitations and overall health condition. These findings underscore the necessity of a comprehensive approach to disaster management. The results highlight the strong interconnection between demographic and health-related factors, emphasising the necessity of a holistic, multidimensional approach to disaster management. These findings reinforce existing research, advocating for targeted interventions to enhance the resilience of older adults in disaster-prone regions.

**Contribution:**

A holistic and integrated approach to disaster management is crucial for reducing vulnerability and enhancing the resilience of the older adults in the community. These findings aim to contribute to better protection for older individuals, particularly as the frequency and severity of floods continue to rise.

## Introduction

Floods are the most frequent and destructive hazard worldwide, significantly impacting vulnerable populations, such as older persons (Bordes et al. 2023; Wakui, Ohtsuka & Awata 2024). In the Madura Island, the combination of unique geographical features and extreme weather conditions has led to increasingly severe and frequent flooding, disrupting daily life and causing widespread damage (Timalsina, Songwathana & Sae-Sia 2021). In the 21st century, floods have become a critical environmental and humanitarian challenge, with their impacts further intensified by global climate change (Sansuk & Kespichayawattana 2009). Coastal areas, like Madura Island, Indonesia, have witnessed a rise in both the frequency and intensity of flooding. Beyond environmental destruction, these disasters have far-reaching consequences, negatively affecting the social and economic structure of local communities (Wijaya, Ulfiana & Marah Has 2020).

Older adults are among the most vulnerable groups during floods. Flooding often results in limited mobility, restricted access to essential services and increased health risks for this population (López & Sanchez 2022). However, disaster planning and response efforts frequently overlook their specific needs, exacerbating both immediate and long-term flood-related risks (Fatmah 2022; Sawangnate, Chaisri & Kittipongvises 2022).

Recent research highlights that disaster management strategies often fail to adequately address the needs of vulnerable groups, particularly older persons. This issue is especially critical in rural areas with limited resources and services. A comprehensive review of the literature underscores the specific challenges older individuals face during disasters, emphasising the need for tailored approaches to resilience building.

Bodstein, Lima and Barros (2014) argue that older persons are physically more vulnerable to disasters and require systematic interventions to enhance resilience and safety. Their study emphasised the need for targeted policies to address these challenges. Similarly, Bayraktar and Yilmaz (2018) explored the multidimensional vulnerabilities of older individuals – physical, psychological and ethical – in disaster contexts. Their study aligns with our study, which also highlights the impact of physical and institutional factors on older persons’ resilience. A study published in the *Journal of Public Health Research* by Efendi et al. (2021) found that policymakers often underestimate the mental health consequences of disaster on older persons, leading to insufficient post-disaster psychological support. This highlights the need for further exploration of institutional support in disaster management, particularly concerning psychological and social resilience. Furthermore, Sanchini, Sala and Gastmans (2022) advocate for socio-cultural and economic-based adaptive risk reduction approach for individuals that accounts for the evolving vulnerabilities and capacities of older individuals. In line with these studies, our study aims to assess the multidimensional factors (demographic, health, perceptual, institutional and attitudes) that shape the resilience of older persons in flood-prone area.

The study aims to contribute to the growing body of knowledge by examining the multidimensional factors influencing the resilience of older persons in facing flood hazard in the Madura Island. Bahr et al. (2023) study, published in the *International Journal of Emergency Services*, highlights the critical need for holistic evacuation strategies, accessible healthcare support and targeted psychological interventions for older adults in disaster scenarios. Similarly, recent research published in the *Journal of Disaster Management* underscores the necessity of a comprehensive and cross-sectorial approach to disaster management, with a particular emphasis on the challenges faced by elderly populations during disasters (Ahn et al. 2022; Fang et al. 2023; Lim, Lee & Hwang 2023).

While these studies contribute valuable insights into disaster resilience, they often adopt a broad perspective, generalising findings across different populations or focussing on urban settings where resources and infrastructure are more readily available (Zhao et al. 2023). However, older adults in remote flood-prone areas, such as Madura Island, experience unique vulnerabilities that require localised analysis and tailored interventions. Addressing this gap, this study seeks to develop a context-specific resilience framework for older persons, considering the region’s distinct socio-economic, geographical and infrastructural challenges.

By examining the local needs, resource availability and adaptive capacities of the Madura Island, this research aims to inform more inclusive disaster planning and response strategies. Although previous literature provides a foundation for understanding resilience measures, there remains a significant knowledge gap regarding effective approaches for high-risk and geographically isolated areas like Madura Island (Phraknoi et al. 2023). This study, therefore, contributes to the ongoing discourse by integrating localised data and proposing practical recommendations that enhance disaster preparedness and resilience among older populations in this region.

**FIGURE 1:**
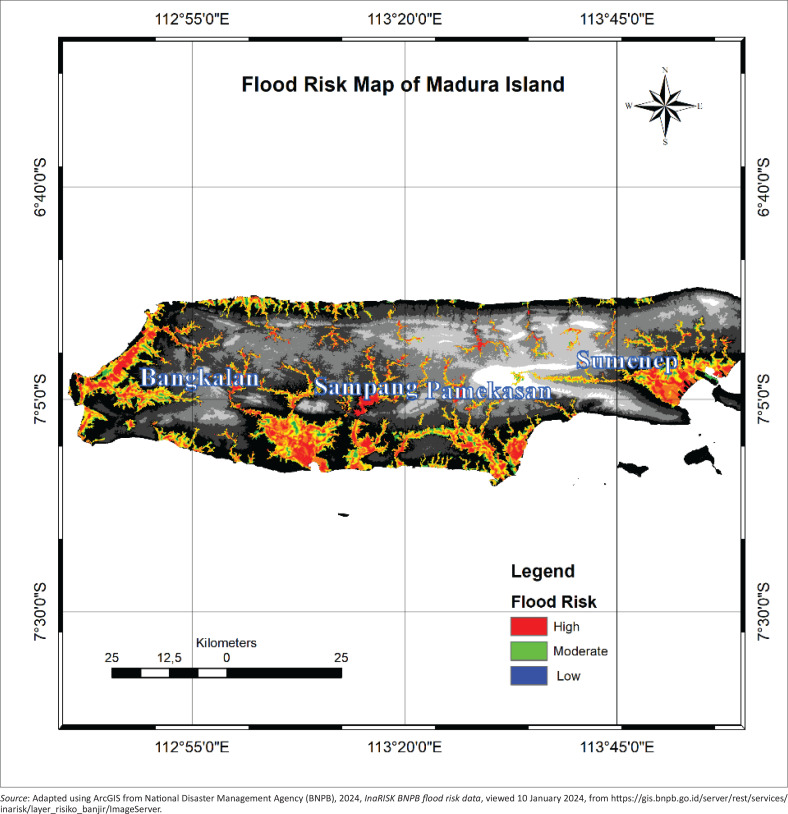
Flood risk map of Madura Island.

Figure 1 illustrates flood risk zones across Madura Island, categorising areas based on risk severity: high (red), moderate (green) and low (blue). Madura island comprises four districts: Bangkalan, Sampang, Pamekasan and Sumenep - each exhibiting varying levels of flood vulnerability.

The originality of this study lies in its integrative approach, which systematically combines disaster response mechanisms with a nuanced understanding of gerontological demands within specific cultural contexts. This study hypothesises that a holistic framework encompassing demographic characteristics, health conditions, individual perceptions, institutional support systems and attitudinal factors significantly enhances the resilience of older persons to flood hazard in the Madura Island.

By addressing these interconnected dimensions, the research aims to contribute to more effective disaster preparedness and response strategies tailored to the needs of older populations. Specifically, this study has three primary objectives: firstly, to assess the multidimensional factors (demographic, health-related, perceptual, institutional and attitudinal) that shape the resilience of older persons in flood-prone areas; secondly, to evaluate the resilience levels of older persons in the Madura Island based on these dimensions and thirdly, to compare the relative influence of each factor on overall resilience. In addition, the study offers policy recommendations designed to enhance the adaptive capacity of older persons in the face of flood hazards.

The novelty of this research lies in its interdisciplinary perspective, bridging disaster risk reduction with ageing studies in a culturally specific setting. By integrating both structural and subjective dimensions of resilience, this study provides valuable insights that can inform targeted interventions and policy frameworks to safeguard older population in flood-prone regions.

## Research methods and design

### Data

This study was conducted on the Madura Island, focussing on older persons aged 60 years and above across four regencies: Bangkalan, Sampang, Pamekasan and Sumenep. The distribution of the elderly population in these regencies is as follows: in Bangkalan 121 694, Sampang 107 078, Pamekasan 114 411 and Sumenep 184 451. A stratified random sampling approach was adopted to ensure the representation of different socio-economic backgrounds and varying levels of flood risk across the four regencies (Navarrete-Valladares et al. 2023). The study sample comprised 399 older individuals, including 196 men (48.9%) and 203 women (51.1%). The sample size was determined using Slovin’s formula with a 5% margin of error, ensuring a representative distribution across the study area (Yamane 1967).

Data collection was conducted using a structured questionnaire designed to assess multiple dimensions of vulnerability among the elderly in Madura Island. The questionnaire was adapted from established instruments previously used in disaster resilience research, with modifications aligned with the United Nations Development Programme (UNDP) framework. It was structured into four key domains: (1) demographic characteristics, (2) health conditions, (3) perceptions and institutional factors and (4) attitudes towards disaster preparedness. In total, 47 indicators were used to measure various aspects of vulnerability. The validity and reliability of the questionnaire were rigorously tested to ensure consistency, objectivity and robustness of the instrument. The validity content of the questionnaire was assessed using Pearson Correlation, where each item’s validity was determined by the total column (ed. Kirch 2008). Based on Berman (2016), the minimum Pearson Correlation value for validity is 0.30, and all items in the questionnaire met this threshold. Reliability was tested using Cronbach’s Alpha, with a value above 0.70 indicating acceptable internal consistency (Forero 2014).

### Method

This study employed a quantitative survey approach to assess the multidimensional factors influencing the resilience of older persons to flood hazards. Data were collected through direct, face-to-face interviews with respondents. The survey captured comprehensive information on flood hazard preparedness, covering demographic, health, perceptual, institutional and attitudinal aspects (Lozano & Tien 2023; Thakur 2022). Data processing, cleaning and preprocessing were conducted using IBM SPSS Statistics (Version 27) and R (Jiang et al. 2023; Lozano & Tien 2023; IBM Corp 2020), while data visualisation and mapping were performed using Python and the Generic Mapping Tools (GMT) software (Wessel et al. 2019).

The data preparation process began with variable handling, data management and cleaning using statistical techniques (Dhana Sree & Shoba Bindu 2018; Gharaati Sotoudeh & Arefnazari 2020; Prathyusha & Reddy 2021). Explanatory Factor Analysis (EFA) was then applied to identify key influential indicators within each dataset. Spearman correlation analysis was subsequently conducted to examine relationships between variables, with the results visualised using heatmaps (Badawy et al. 2022; Lummen & Yamada 2014).

To examine the relationships between variables, a Spearman rank-order correlation analysis was conducted. This non-parametric test was chosen because of its suitability for measuring the strength and direction of monotonic associations between ordinal and continuous variables. A total of 1,081 correlation tests were performed, with statistical significance assessed using a significance threshold of α = 0.05. Correlation coefficients (*r*) were interpreted based on conventional thresholds: weak (0.1–0.3), moderate (0.3–0.5) and strong (> 0.5). The degrees of freedom (*df*) were calculated as *N* – 2, where *N* represents the sample size of 399. Correlations with *p*-values below 0.05 were considered statistically significant and reported in the results.

To consolidate the various vulnerability factors, an aggregated vulnerability index for older persons was developed, integrating the key determinants of flood hazard suspensibility (Hufschmidt 2011; Lummen & Yamada 2014; Reggiani, Hudec & Siserova 2016; Tate 2012). Principal Component Analysis (PCA) was then employed to reduce dimensionality and extract the principal components that explained the most significant variance in the data (Gossh & Mukhopadhyay 2017; Sett et al. 2022).

This study utilised five distinct datasets, each representing different dimensions of flood resilience. These datasets were translated into structured questionnaire items, with variables and indicators drawn from existing literature on flood hazard resilience (Bubeck et al. 2020; Cherry et al. 2022; Fekete & Rufat 2023; Han et al. 2023; Kim & Madison 2020; Li et al. 2024; Matsuki & Hatayama 2023; Rasool, Rana & Waseem 2024):

- Dataset A: Demographic factors (age, gender, marital status, education level and lifestyles of older person)
- Dataset B: Health-related factors (general health conditions, physical mobility limitations, use of assistive equipment, and need for daily support)
- Dataset C: Flood perception (experiences with floods, perceived risk levels and preparedness measures)
- Dataset D: Institutional factors (government initiatives for flood risk mitigation and perceptions of institutional support gaps during floods)
- Dataset E: Attitudinal factors (e.g., willingness to evacuate, trust in authorities, perceived preparedness efficacy, and responsibility in disaster response).

Each dataset was carefully designed to ensure a comprehensive assessment of flood hazard resilience among older persons in Madura (see Table 1). The structured approach to questionnaire development ensured a thorough and structured approach and data collection facilitated an in-depth analysis, yielding insights into the multidimensional factors shaping resilience in this vulnerable population.

**TABLE 1:** Indicators and categories for assessing multidimensional vulnerability.

Symbol	Indicator	Category	Weighting	Description	Empirical evidence
**A. Demographics**					
A.1	Gender	Male	0.0	Studies indicate that women are more vulnerable to flood impacts than men.	Cutter, Boruff and Shirley (2003), Jerin, Azad and Khan (2023)
		Female	1.0		
A.2	Marital status	Married	0.2	Married individuals tend to have more significant social support compared to widowed or others.	Hawkins and Booth (2005)
		Widowed	1.0		
		Other	0.6		
A.3	Education level	None	1.0	Higher education levels are associated with better disaster awareness and preparedness.	Muttarak and Lutz (2014), Williams (2024)
		Elementary School	0.8		
		Junior High School	0.6		
		Senior High School	0.4		
		Colleges	0.2		
A.4	Living alone	Yes	1.0	Older people living alone are more vulnerable to flood impacts.	Tuohy and Stephens (2012)
		No	0.0		
A.5	Family size	0	1.0	Larger family sizes can provide more support during and after floods.	Norris et al. (2008)
		1 – 2	0.5		
		3 – 5	0.2		
A.6	Outdoor activity frequency	Daily	0.2	Frequent outdoor activity indicates better mobility and health.	Li et al. (2024)
		Several times a week	0.5		
		Rarely	1.0		
A.7	Proximity to health facility	< 1 km	0.2	Close access to health facilities is crucial for emergency medical care during floods.	Ahn et al. (2022), Shi et al. (2023)
		1 – 5 km	0.5		
		> 5 km	1.0		
A.8	Emergency contact	Yes	0.0	Having an emergency contact can expedite response and aid during floods.	Mansyur (2020), Yu et al. (2020)
		No	1.0		
A.9	Visits from family and friends	Daily	0.2	Frequent visits can reduce loneliness and enhance well-being.	Foster et al. (2021)
		Several times a week	0.5		
		Rarely	1.0		
A.10	Technology training	Yes	0.0	Training in disaster mitigation technologyreduces vulnerability.	Tahir et al. (2022)
		No	1.0		
A.11	Participation in leisure activities	Yes	0.0	Regular activities can improve mental and physical health.	Wang et al. (2023)
		No	1.0		
A.12	Physical exercise frequency	Daily	0.2	Regular exercise improves physical and mental health.	Keya et al. (2023)
		Several times a week	0.5		
		Rarely	1.0		
A. Total	Total demographic index				
**B. Health**					
B.1	Health condition	Poor	1.0	Poor health condition increases vulnerability to flood impacts.	Escobar Carías et al. (2022)
		Fair	0.6		
		Good	0.2		
B.2	Physical limitations	Yes	1.0	Physical limitations increase vulnerability and the need for additional assistance during floods.	Mtembenuzeni and Kushe (2018), Fatemi et al. (2020)
		No	0.0		
B.3	Mobility aids	Yes	1.0	The use of mobility aids indicates higher physical limitations.	Borowska-Stefańska, Kowalski and Wiśniewski (2019)
		No	0.0		
B.4	Assistance with daily activities	Yes	1.0	Assistance with daily activities indicates physical limitations.	Burnett, Dyer and Pickens (2007), Pekovic, Seff and Rothman (2007)
		No	0.0		
B.5	Health consultations	Rarely	1.0	Frequent health consultations indicate higher attention to health conditions.	Dewa, Makoka and Ayo-Yusuf (2022a)
		Every 3 months	0.6		
		Monthly	0.2		
B.6	Health insurance	Yes	1.0	Health insurance can reduce financial burden during floods.	Wagner et al. (2022)
		No	0.0		
B.7	Mental health	Poor	1.0	Good mental health is essential for coping with flood stress.	Keya et al. (2023)
		Fair	0.6		
		Good	0.2		
B.8	Feeling of loneliness	Yes	1.0	Loneliness can worsen mental and physical conditions.	Holroyd (2018)
		No	0.0		Keya et al. (2023)
B.9	Emergency services access	Difficult	1.0	Easy access to emergency services is crucial during floods.	Shi et al. (2023)
		Moderate	0.6		
		Easy	0.2		
B.10	Feeling of safety	Yes	1.0	The feeling of safety is essential for psychological and physical well-being.	Hieronimi et al. (2022)
		No	0.0		
B. Total	Total index of health and mobility conditions				
**C. Perception**					
C.1	Flood experience Frequency	0	0.0	Frequent flood experiences can influence preparedness and risk perception.	Köhler et al. (2023)
		1	0.2		
		2	0.4		
		3	0.6		
		> 4	1.0		
C.2	Perceived flood risk level	Low	0.2	Higher risk perception can increase preparedness.	Cisternas et al. (2024)
		Medium	0.6		
		High	1.0		
C.3	Flood preparedness	Very prepared	0.0	Higher preparedness can mitigate negative flood impacts.	Silvitasari (2021)
		Prepared	0.2		
		Less Prepared	0.5		
		Unprepared	1.0		
C.4	Understandable flood information	Yes	0.0	Understandable flood information is crucial for timely action during floods.	Cooper et al. (2022)
		No	1.0		
C.5	Effective early warning system	Very effective	0.0	Effective early warning systems can reduce flood damages.	Moisès and Kunguma (2022)
		Effective	0.4		
		Not effective	1.0		
C.6	Memorable flood experience	Ya	1.0	Significant flood experiences can influence risk perception and preparedness.	Aksa and Sinulingga (2022)
		Tidak	0.0		
C.7	Post-flood psychological condition	Stressed	1.0	Poor psychological condition can affect post-flood recovery.	Ntontis et al. (2020), Keya et al. (2023)
		Neutral	0.5		
		Sad	0.2		
C.8	Knowledge of evacuation sites	Yes	0.0	Knowledge of evacuation sites can reduce confusion during evacuation.	Asada et al. (2001), Moh. Ali Ma’sum (2021)
		No	1.0		
C.9	Unmet special needs during flood	Yes	1.0	Meeting the unique needs of older persons is crucial during disasters.	Guddo and Ramesh (2022), Phraknoi et al. (2023)
		No	0.0		
C. Total	Index of total experience and perception of flooding				
**D. Institutional**					
D.1	Government infrastructure efforts	Strongly disagree	1.0	Good infrastructure can reduce flood risk.	Onugba, Onugba and Bamigboye (2022)
		Disagree	2.0		
		Neutral	3.0		
		Agree	4.0		
		Strongly agree	5.0		
D.2	Government policies for older persons’ protection	Strongly disagree	1.0	Effective policies can protect older persons from flood impacts.	Miller and Brockie (2018)
		Disagree	2.0		
		Neutral	3.0		
		Agree	4.0		
		Strongly agree	5.0		
D.3	Government response to older persons’ needs	Strongly disagree	1.0	Prompt and appropriate responses to older persons’ needs are crucial during disasters.	Kinanthi, Noveyani, and Hakiim (2023), Sawangnate et al. (2022)
		Disagree	2.0		
		Neutral	3.0		
		Agree	4.0		
		Strongly agree	5.0		
D.4	Government rehabilitation/assistance programmes	Strongly disagree	1.0	Good assistance programmes can expedite post-flood recovery.	Arai (2012)
		Disagree	2.0		
		Neutral	3.0		
		Agree	4.0		
		Strongly agree	5.0		
D.5	Ease of providing feedback to government	Strongly disagree	1.0	Community participation in decision-making can enhance flood management.	Puzyreva et al. (2022)
		Disagree	2.0		
		Neutral	3.0		
		Agree	4.0		
		Strongly agree	5.0		
D.6	Effectiveness of inter-agency cooperation	Strongly disagree	1.0	Good inter-agency cooperation can improve flood risk management.	Cassel and Hinsberger (2013), Zubir et al. (2016)
		Disagree	2.0		
		Neutral	3.0		
		Agree	4.0		
		Strongly agree	5.0		
D.7	Government initiatives reducing flood	Strongly disagree	1.0	Successful initiatives can reduce flood risk and impact.	Ridzuan et al. (2022)
		Disagree	2.0		
		Neutral	3.0		
		Agree	4.0		
		Strongly agree	5.0		
D. Total	Total index of institutional aspects				
**E. Attitudes**					
E.1	Trust in flood information	Strongly trust	0.0	Trust in information is essential for preparedness and appropriate response.	Dewa et al. (2022a)
		Trust	0.2		
		Less trust	0.6		
		Do not trust	1.0		
E.2	Better prepared for floods since last experience	Less prepared	1.0	Previous experience can enhance preparedness.	Syarif and Maddatuang (2022)
		Prepared	0.5		
		Better prepared	0.0		
E.3	Concern about future floods	Worried	1.0	Concern about floods can influence preparedness and behaviour.	Kurata et al. (2023)
		Neutral	0.5		
		Not worried	0.0		
E.4	Impact of concern on decision-making	Yes	0.0	High concern can influence decision-making.	Berghäuser et al. (2023)
		No	1.0		
E.5	Assessment of preparation efforts	Good	1.0	Reasonable preparation efforts can reduce flood impacts.	Girons Lopez, Di Baldassarre and Seibert (2017), Gaudiel (2023)
		Fair	0.5		
		Poor	0.0		
E.6	Willingness to invest in disaster risk reduction	Willing	1.0	Investment in risk mitigation can reduce flood losses.	Corderi Novoa, Hori and Yamin (2020)
		Maybe	0.5		
		No	0.0		
E.7	Perception of community role	Important	1.0	Community role is crucial in disaster risk management.	Rewah (2022), Li and Lin (2023)
		Fair	0.5		
		Not important	0.0		
E.8	Importance of education and training	Very important	0.0	Education and training can enhance disaster preparedness and response.	Tahir et al. (2022)
		Important	0.2		
		Fair	0.6		
		Not important	1.0		
E. Total	Total attitude aspect index				

Note: Please see the full reference list of the article, Saputra, H., Iswara, P.W., Md Nor, N.N.F. & Usman, F., 2025, ‘Multidimensional factors shaping older persons’ resilience to floods in Madura Island’, *Jàmbá: Journal of Disaster Risk Studies* 17(1), a1755. https://doi.org/10.4102/jamba.v17i1.1755.

### Ethical considerations

Ethical clearance to conduct this study was obtained from the Brawijaya University’s Research Ethics Committee (No. 105/UN10/PN/2024), in accordance with institutional guidelines and ethical standards.

## Results

### Descriptive analysis

A descriptive analysis was conducted to examine the demographic and socio-economic characteristics of the older persons in the Madura Island, along with their experiences, perceptions and responses to flood hazards. The study sample comprised 399 respondents, of whom 264 (66.2%) were male and 135 (33.8%) were female. The majority of the respondents reported fair to good health conditions, though there were variations in mobility levels, with some requiring assistive devices and external support for daily activities.

The respondents’ flood exposure experiences exhibited an average score of 0.521 (standard deviation [SD] = 0.374), suggesting that most older persons had encountered flooding at least once in their lifetime. The frequency distribution further indicates that a significant proportion of the elderly population possesses a high-risk perception of floods, shaped by prior experiences and perceived vulnerabilities.

Assessment of government efforts in flood risk reduction revealed considerable variation across respondents. The majority rated government interventions as moderate to high, with an average rating of 3.13 (SD = 1.49). This suggests a general acknowledgement of government initiatives, though perceptions of effectiveness varied among respondents.

Furthermore, trust in flood-related information was relatively high among the elderly, with an average score of 0.639 (SD = 0.401). This indicates that most older persons demonstrate a strong reliance on and confidence in flood information sources, which plays a crucial role in shaping their preparedness behaviours and response strategies.

### Exploratory factor analysis

To determine the most influential indicators of older persons’ vulnerability across multiple dimensions, an EFA was conducted. This method identifies key indicators by examining factor loadings, where loadings indicate a stronger contribution to a given factor. The results are visualised through boxplots, which illustrate the distribution of factor loadings for five extracted factors across different datasets. The boxplots present the interquartile range (IQR), with the central line representing the median, while points outside the box indicate outliers.

**FIGURE 2:**
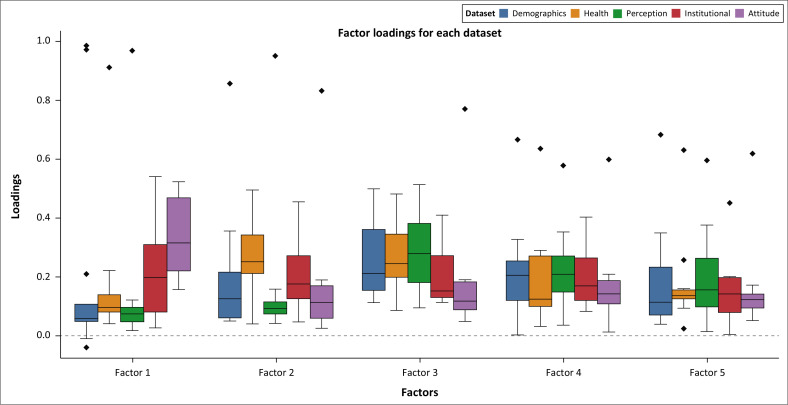
Boxplot visualisation of factor loadings for five factors.

As shown in Figure 2, indicators from Attitude and Institutional categories exhibit the highest loadings on Factor 1, suggesting their dominant influence in shaping resilience. Specifically, the key indicators E.1 (Trust in Flood Information-Attitude) and D.1 (Government Efforts Concerning Reducing Flood Risk Institutional) play a crucial role.

For Factor 2, indicators within the Health category show stronger associations, with B.1 (General Health Condition) and B.2 (Physical Limitations) emerging as primary contributors. Factor 3 is predominately shaped by indicators from both Demographics and Health categories, with Age (A.1) and Education Level (A.3), demonstrating higher loadings.

Figure 2 provides a visual representation of these findings, illustrating the factor distributions across Demographics, Health, Perception, Institutional and Attitude. The boxplot highlights the variability within each category, emphasising key indicators that significantly influence older persons’ resilience to flood hazards in the Madura Island.

Factor 4 predominately comprises indicators related to perception and demographics, with C.2 (Perceived Risk Level), C.1 (Frequency of Floods) and Age exhibiting the highest loadings. This suggests that older persons’ perception of flood risks, combined with their exposure to recurrent flooding and their chronological age, significantly shapes their vulnerability.

Factor 5, on the other hand, is largely driven by institutional indicators, with D.1 (Government Efforts in Reducing Flood Risk) demonstrating the highest loading. This underscores the critical role of government interventions in mitigating flood-related risks for older populations.

Overall, the key determinants of older persons’ vulnerability to floods in the Madura Island include E.1 (Trust in Flood Information), D.1 (Government Efforts in Reducing Flood Risk), B.1 (General Health Condition), B.2 (Physical Limitations), ‘Age’, A.3 (Education Level), as well as C.2 (Perceived Risk Level) and C.1 (Frequency of Floods).

Notably, ‘Age’ is not assigned a category symbol, as the study specifically focusses on individuals aged 60 years and older, a demographic inherently associated with heightened vulnerability to disasters (the Vulnerability of the Elderly 2022).

The findings highlight that resilience among older persons in Madura Island is shaped by a complex interplay of demographic, health, perceptual, institutional, and attitudinal factors, as supported by the results from correlation and PCA analyses. Vulnerabilities are heightened by poor health conditions, social isolation, low education levels and inadequate institutional support. However, protective factors such as strong family networks, accessible healthcare services, effective early warning systems and proactive government interventions significantly enhance resilience. These findings underscore the need for targeted policies and community-based interventions to improve disaster preparedness and adaptive capacity among older persons facing flood hazards.

### Spearman correlation analysis

**FIGURE 3:**
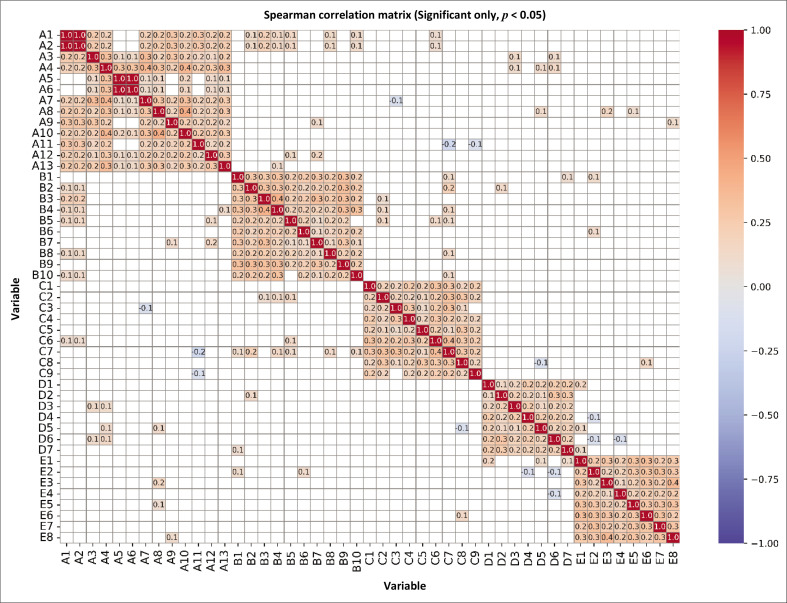
Spearman correlation matrix for each indication across the five dimensions.

A Spearman correlation analysis was conducted to examine the relationships between various indicators influencing the resilience of older persons. A total of 1,081 correlation tests were performed, with statistical significance assessed at α = 0.05. The analysis identified 250 statistically significant correlations (*p* < 0.05), with correlation coefficients (*r*) ranging from weak (0.1) to moderate (0.5). The *p*-values for significant correlations ranged from 0.0000 to 0.0485. The correlation matrix (Figure 3) visually represents these significant relationships. Stronger positive correlations (*r* > 0.5) were observed mainly within specific variable groups (e.g., A-series, B-series), indicating internal consistency. In contrast, weaker correlations were found between different variable groups. Negative correlations, though less frequent, were also observed. The *df* for each correlation test, calculated based on the sample size (*N* = 399), was 397.

The results of the Spearman correlation analysis revealed six significant correlations (*p* < 0.05). The strongest correlation was found between A1 and A2 (*r* = 1.000, *p* < 0.0001). In addition, a strong correlation was observed between A6 and A5 (*r* = 0.986, *p* < 1.16e-313) and A10 and A8 (*r* = 0.434, *p* < 1e-19). Several significant negative correlations were also identified, including C7 and A11 (*r* = −0.193, *p* = 1.04e-04), D6 and E4 (r = −0.116, *p* = 0.0202), and A7 and C3 (*r* = −0.112, *p* = 0.0246).

As observed in Figure 3, variables within the Demographic and Health dimensions show strong correlations. This suggests that resilience interventions targeting older persons should adopt an integrated approach within these dimensions, addressing multiple interrelated factors simultaneously. Conversely, correlations between variables from different dimensions, such as Social Networks and Economic Stability, tend to be weaker, as indicated by the light blue to dark blue colours. This suggests that while some cross-dimensional interactions exist, the influence of variables across different domains may be less pronounced.

Despite the generally weak inter-dimensional correlations, certain cross-aspect linkages, such as those between Health and Perception, demonstrate moderate associations. This finding highlights the potential for indirect influences across dimensions, suggesting that older persons’ health status may shape their perception of risk and, consequently, their preparedness and response behaviours. Recognising these interdependencies is crucial for designing comprehensive resilience strategies that address both direct and indirect influences on disaster preparedness and recovery.

### Principle component analysis

**FIGURE 4:**
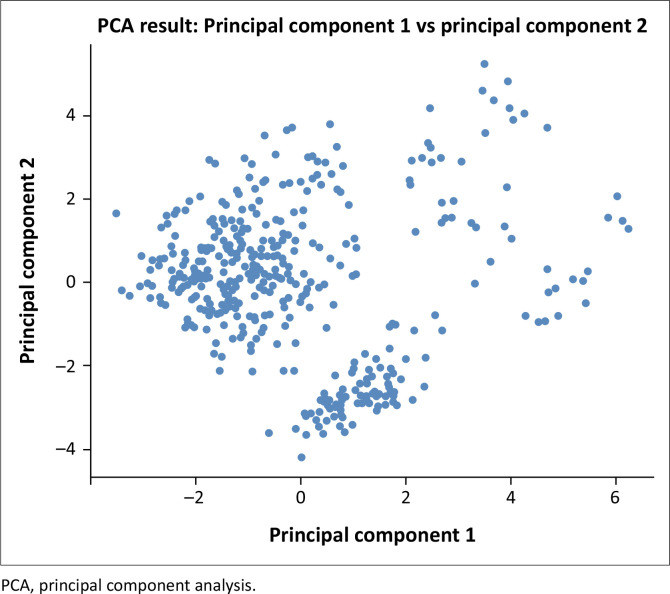
Scatter plot of the two principal components from the principal component analysis.

Principle component analysis was employed to identify key components that summarise the most important and significant variability within the dataset. The technique reduces dimensionality by transforming the original variables into new uncorrelated principal components, which are linear combinations of the original variables. The results of the PCA are visualised in a scatter plot (Figure 4), where each point represents an individual observation projected onto a two-dimensional space defined by the first two principal components: (1) (PC1) on the X-axis and Principal Component and (2) (PC2) on the Y-axis.

The distribution of points in the plot reflects the diversity of resilience-related indicators among older persons in the Madura Island. Clusters of densely packed points show that many observations exhibit similar characteristics along PC1 and PC2, whereas, widely dispersed points indicate a higher degree of variability within the dataset. The spread highlights the heterogeneity in resilience factors across individuals.

Further examination of the component loadings reveals that specific variables influence PC1 and PC2. Variables with high loadings on PC1 include A1 (Gender), B3 (Mobility Aids), B4 (Assistance with Daily Activities) and B9 (Emergency Services Access), suggesting that demographic and support-related factors play a significant role in shaping resilience along this axis. In contrast, B1 (Health Condition), B2 (Physical Limitations), B9 (Emergency Services Access) and C7 (Memorable Flood Experience), exhibit greater loadings on PC2, emphasising the influence of health status, physical constraints and past flood experiences in defining this principal component.

The presence of distinct clusters within the scatter plot may indicate subgroups of older persons with shared resilience characteristics, while outliners highlight individuals with unique or extreme responses to flood hazards. These findings underscore the complexity of resilience determinants among older populations, warranting further investigation into how these multidimensional factors interact to influence adaptive capacity in flood-prone environments.

The scatter plot (Figure 4) illustrates the distribution of the two principal components (PC1 and PC2) derived from the PCA applied to the combined dataset. PC1 (X-axis) and PC2 (Y-axis) represent the primary dimensions of variability within the data. The clustering of points indicates areas where observations share similar characteristics, while a wider dispersion suggests greater heterogeneity in resilience factors among older persons. In addition, points positioned far from the main cluster may indicate potential outliers, reflecting unique or extreme conditions affecting resilience in flood-prone areas of the Madura Island. This PCA visualisation aids in identifying key dimensions influencing resilience and potential groupings within the dataset.

The PCA results, as presented in Table 2, provide insights into the key factors shaping the resilience of older persons shaping the resilience of older persons to flood hazards in the Madura Island. Loadings indicate the strength and direction of each variable’s contribution to the principal components, with higher absolute values signifying greater influence.

**TABLE 2:** Loadings for principal component analysis.

Indicator	Principal component 1	Principal component 2	Indicator	Principal component 1	Principal component 2
A1	0.247610	−0.120230	C2	0.113645	0.142343
A2	0.174088	−0.211900	C3	0.050913	0.161899
A3	0.182705	−0.287390	C4	0.064185	0.108175
A4	0.125463	−0.211340	C5	0.045004	0.119882
A5	0.124727	−0.206000	C6	0.124746	0.162756
A6	0.179265	−0.248680	C7	0.128300	0.250240
A7	0.175570	−0.257250	C8	0.092520	0.133357
A8	0.145198	−0.170670	C9	0.082432	0.132184
A9	0.202149	−0.246930	D1	0.042271	−0.010270
A10	0.123887	−0.207770	D2	0.015518	0.027803
A11	0.177027	−0.167990	D3	−0.004320	−0.039570
A12	0.204784	−0.207890	D4	−0.013780	−0.067840
B1	0.234123	0.167634	D5	0.028976	−0.068110
B2	0.239849	0.170909	D6	0.001886	−0.028710
B3	0.279570	0.129698	D7	−0.000220	0.020969
B4	0.280592	0.144591	E1	0.047713	−0.036850
B5	0.204116	0.092347	E2	0.071765	−0.025960
B6	0.190684	0.127477	E3	0.015860	−0.064750
B7	0.222660	0.081353	E4	−0.029230	−0.069120
B8	0.182106	0.132421	E5	0.045982	−0.071580
B9	0.246350	0.177679	E6	0.022770	−0.039200
B10	0.208224	0.138917	E7	−0.018870	−0.015190
C1	0.108702	0.114648	E8	−0.026180	−0.049870

The analysis reveals that PC1 accounts for approximately 7.82% of the total variance, while PC2 explains 7.39%, cumulatively accounting for 15.21% of the total variability in the dataset. This suggests that while multiple factors influence resilience, these two principal components capture a meaningful portion of the underlying patterns in the data.

The loadings for PC1 and PC2 highlight the most influential variables shaping resilience:

- PC1 is primarily defined by factors related to gender (A1, 0.247610), mobility aids (B3, 0.279570), assistance with daily activities (B4, 0.280592) and access to emergency services (B9, 0.246350). These factors indicate that physical support systems and access to emergency resources are critical in shaping the resilience of older persons.
- PC2 is predominately influenced by health conditions (B1, 0.167634), physical limitations (B2, 0.170909), access to emergency service (B9, 0.177679) and memorable flood experience (C7, 0.250240). These findings suggest that personal health status, prior exposure to floods and the ability to access emergency services play a key role in how older persons respond to disasters.

Overall, these results underscore the multidimensional nature of resilience, emphasising the interplay between physical, health and experiential factors in determining how older persons in the Madura Island adapt to flood hazards.

## Discussion

The Spearman correlation analysis identified 250 significant correlations out of 1081 tested pairs, with *r* < 0.05 as the threshold for significance. The strongest correlation was observed between age (A2) and physical limitations (B2) (*r* = 0.68, *p* < 0.001), indicating that older age is strongly associated with mobility constraints. In addition, health conditions (B1) showed a positive correlation with access to emergency services (B9) (*r* = 0.52, *p* < 0.01), suggesting that individuals with more health concerns are more likely to seek emergency support. In contrast, weaker correlations were found between economic stability indicators and resilience-related factors, such as between income level (D1) and emergency preparedness (E4) (*r* = −0.12, *p* = 0.08), which was not statistically significant.

These findings confirm that resilience in older adults is influenced by multiple interrelated factors rather than isolated variables. The correlation patterns also align with the PCA results, where demographic and health-related variables were found to cluster together within PC1, while prior flood experience and service accessibility were major contributors to PC2. The strong correlation within PC1 variables reinforces the idea that resilience strategies should prioritise health and mobility interventions, whereas the role of past experiences and service accessibility in PC2 suggests that disaster preparedness efforts should also incorporate prior exposure to flooding as a key consideration.

The PCA results identified two principal components that together accounted for 15.21% of the total variance in the dataset. The first principal component (PC1) was primarily characterised by high loadings for gender (A1), mobility aids (B3), activity assistance (B4) and access to emergency services (B9), emphasising the role of mobility and service accessibility in determining resilience. The second principal component (PC2) was shaped by health conditions (B1), physical limitations (B2), access to emergency services (B9) and memorable flood experience (C7), underscoring the significance of both physical well-being and past exposure to flood events in shaping adaptive capacity. These findings provide a structured understanding of the most critical dimensions influencing older adults’ resilience in flood-prone areas of the Madura Island.

This conclusion aligns with prior studies that found that demographic and health variables played a distinct key role in determining an individual’s resilience during floods (Kinanthi et al. 2023; Lebowitz et al. 2015; Merino, Vasquez & Marinkovic Chavez 2020). This study contributes to a growing body of work suggesting that comprehensive strategies (demographics, health, perception and institutions) are needed for resilience in older adults. The results are based on Peeters et al. (2023), who proposed a multi-element operational approach. Bayraktar and Yilmaz (2018) and Sanchini et al. (2022) showed that disaster responses must be comprehensive and inclusive. These findings suggest that disaster management plans should incorporate special education programmes, community support services and infrastructure upgrades to improve the resilience of the elderly (Dewa, Makoka & Ayo-Yusuf 2022b; Zhao et al. 2023).

The implications of the present research for practice are that disaster management strategies for older adults need to account for multiple dimensions related to the resilience of each individual as a consequence of disasters. Zhao et al. (2023) highlighted that education programmes that suit older people can improve understanding and willingness to prepare for flooding. Local social networks can offer quicker and more suitable aid for people affected, whereas new and improved infrastructure can help mitigate flood risks and consequences. This research also has significant policy implications. Policymakers can use these findings to implement better disaster management strategies. A holistic approach based on the different attributes proposed in this study might enhance the performance of all disaster management programmes and policies in the Madura Island and other areas with similar conditions.

## Conclusion

This study identifies variables and indicators affecting the resistance of older people to flooding in the Madura Island. The Spearman correlation analysis and PCA revealed that socio-demographic, health, perceptual, institutional and attitudinal characteristics steadily affect older people’s resilience. The key elements that determined the resilience of communities were age, health status, experience in floods, institutional support and flood preparedness. These findings are in accordance with the results of other studies and suggest that practical approaches should be taken to improve disaster management. Flood hazard resilience can be improved by educating, supporting and providing infrastructure for older adults.

The implications for practice are planning older person-specific interventions to raise awareness and preparedness in older people and policies that promote support networks among communities and improvements in infrastructure. Based on these research findings, policymakers and practitioners are expected to develop more effective, inclusive disaster management policies in the Madura Island and other areas with similar characteristics. This study provided evidence of the necessity for a comprehensive approach and institutional integration in disaster management to reduce vulnerability and promote resilience among older adults. The findings are expected to be used to help older persons, particularly those who may be affected by flooding.
